# Spinal Cord Resting State Activity in Individuals With Fibromyalgia Who Take Opioids

**DOI:** 10.3389/fneur.2021.694271

**Published:** 2021-08-04

**Authors:** Katherine T. Martucci, Kenneth A. Weber, Sean C. Mackey

**Affiliations:** ^1^Human Affect and Pain Neuroscience Laboratory, Center for Translational Pain Medicine, Department of Anesthesiology, Duke University Medical Center, Durham, NC, United States; ^2^Systems Neuroscience and Pain Laboratory, Division of Pain Medicine, Department of Anesthesiology, Perioperative, and Pain Medicine, Stanford University, Palo Alto, CA, United States

**Keywords:** chronic pain, opiate, ALFF, low frequency power, cervical spinal cord, fatigue, fMRI, widespread pain

## Abstract

Chronic pain coincides with myriad functional alterations throughout the brain and spinal cord. While spinal cord mechanisms of chronic pain have been extensively characterized in animal models and *in vitro*, to date, research in patients with chronic pain has focused only very minimally on the spinal cord. Previously, spinal cord functional magnetic resonance imaging (fMRI) identified regional alterations in spinal cord activity in patients (who were not taking opioids) with fibromyalgia, a chronic pain condition. Here, in patients with fibromyalgia who take opioids (*N* = 15), we compared spinal cord resting-state fMRI data vs. patients with fibromyalgia not taking opioids (*N* = 15) and healthy controls (*N* = 14). We hypothesized that the opioid (vs. non-opioid) patient group would show greater regional alterations in spinal cord activity (i.e., the amplitude of low frequency fluctuations or ALFF, a measure of regional spinal cord activity). However, we found that regional spinal cord activity in the opioid group was more similar to healthy controls, while regional spinal cord activity in the non-opioid group showed more pronounced differences (i.e., ventral increases and dorsal decreases in regional ALFF) vs. healthy controls. Across patient groups, self-reported fatigue correlated with regional differences in spinal cord activity. Additionally, spinal cord functional connectivity and graph metrics did not differ among groups. Our findings suggest that, contrary to our main hypothesis, patients with fibromyalgia who take opioids do not have greater alterations in regional spinal cord activity. Thus, regional spinal cord activity may be less imbalanced in patients taking opioids compared to patients not taking opioids.

## Introduction

Chronic pain states and opioid medication use both can alter the central nervous system (CNS) via effects on neurophysiologic mechanisms within the brain and spinal cord. While spinal cord mechanisms of chronic pain have been extensively studied in animal models and *in vitro*, to date, research in patients with chronic pain has focused only minimally on the spinal cord. Measurement of spinal cord activity in human chronic pain patients is essential for our understanding of chronic pain because the spinal cord represents the CNS nexus where peripheral inputs, local spinal cord circuits, as well as descending modulatory circuits from supraspinal and brainstem areas all intersect. Further, the spinal cord is a key region where interacting effects would presumably occur from both chronic pain and opioid use. Opioid analgesics exert their pain-relieving effects by acting both locally within the spinal cord dorsal horn and in the brain, which in turn, activates descending inhibition of pain via brainstem to spinal cord projections (1). Thus, investigating the CNS, and specifically the spinal cord, in patients taking opioids may provide insight to how long-term opioid use influences neurophysiology, and thereby provide an additional marker to identify concerns and/or assess appropriateness of opioid use.

Currently, our understanding is limited regarding the effects of long-term opioid use on clinical outcomes in patients with chronic pain. While opioids are a mainstay of perioperative, cancer, and palliative care, the appropriateness of opioid use for long-term treatment of chronic pain is highly debated and controversial. This controversy is due to the potential for adverse effects such as sedation, dizziness, nausea, vomiting, constipation, physical dependence, tolerance, and respiratory depression; as well as the risk for development of opioid use disorder (2, 3). Opioid use is not superior to non-opioid therapy for long-term (i.e., 12-months) treatment of chronic pain (e.g., chronic back pain and knee osteoarthritis) (4). For the chronic pain condition of fibromyalgia, opioid use is particularly controversial, and long-term opioid use vs. non-opioid medication use does not improve physical function or reduce pain interference (5). Patient reports and clinically observed outcomes such as physical function and pain interference inform the limited current understanding of how long-term opioid use affects chronic pain, however, underlying effects of opioid use on neurophysiology remains generally unknown.

Few neuroimaging studies include individuals with chronic pain who take opioids, yet from these studies, it is apparent that opioid-related effects on brain neurophysiology occur rapidly and extensively. For example, structural changes in the brain occur in individuals who take opioids for 1 month for chronic low back pain, and these changes persist for several months after opioid treatment is terminated (6, 7). Similar effects on cortical and subcortical structure and function have been observed in pain-free individuals with opioid use disorder (8). Additionally, chronic pain patients taking opioids show altered frontostriatal functional connectivity (9) and altered brain response to noxious stimulation (10). Further, we have shown that compared to patients not taking opioids, chronic pain patients taking opioids show differential response to reward processing in the brain (11). However, to our knowledge, no studies in patients with chronic pain who take opioid medications have investigated spinal cord activity.

By investigating spinal cord activity, new information can be gained regarding CNS activity in patients with chronic pain who take opioids. Spinal cord activity can be non-invasively and, as demonstrated through technological improvements over the last decade, reliably measured in human clinical research using functional magnetic resonance imaging (fMRI) (12). While fMRI of the brain has been used extensively to identify altered activity within the central nervous system to help elucidate the etiology of the chronic pain condition, fibromyalgia, alterations have also been shown in the periphery (13–15). Thus, to link these peripheral and central nervous system findings, we have extended this evidence to the spinal cord and previously showed regional differences in spinal cord activity in patients with fibromyalgia vs. healthy controls (16). However, none of the patients in the previous study were taking opioid medications. Importantly, individuals with fibromyalgia who take opioids do not tend to do better than their counterparts who take non-opioid medications (5), and use of opioid medications may produce opioid-induced hyperalgesia (2). Opioid-induced hyperalgesia has been documented in clinical populations, and occurs in part, via enhanced spinal cord activity [i.e., increased descending facilitation (17, 18)]. Therefore, we hypothesized that patients with fibromyalgia who take opioids (vs. patients with fibromyalgia who do not take opioids) would show greater regional alterations in spinal cord activity (i.e., enhanced central sensitization).

To test this hypothesis, in the present pilot study, we analyzed resting-state (task-free) fMRI data from the spinal cord in a cohort of patients with fibromyalgia who take opioids. We focused our analysis on the cervical spinal cord based on technological availability (i.e., head and neck coil and fMRI protocol for this region). We compared cervical spinal cord activity (i.e., the amplitude of low frequency fluctuations, ALFF) from the cohort of patients with fibromyalgia who take opioids (i.e., opioid group) to previously analyzed data sets of patients with fibromyalgia who do not take opioids (i.e., non-opioid group) and healthy pain-free controls. Lastly, we tested for behavioral/clinical correlations and compared functional connectivity and graph metrics from the resting-state fMRI data to understand functional network characteristics within the spinal cord that may be differentially altered in patients taking opioids.

## Methods

### Participants

Patients with fibromyalgia not taking opioids (*N* = 17), patients with fibromyalgia taking opioids (*N* = 16) and pain-free healthy controls (*N* = 17) participated in the study. Recruitment and data collection were conducted from May through August 2016. All patients were female and met the following inclusion criteria: modified American College of Rheumatology (ACR) 2011 criteria for fibromyalgia [widespread pain index (WPI) ≥ 7 + symptom severity (SS) ≥ 5, or WPI 3-6 + SS ≥ 9; with symptoms present at the same level for > 3 months; no disorder to otherwise explain the pain] (19), pain in all four body quadrants, average pain over the previous month > 2, not pregnant or nursing, no MRI contraindications, and no depression or anxiety disorder. Patients took their usual medications during the study (Table 1). To reduce potential bias of subject data inclusion within the three groups, the groups were recruited separately using three pre-defined sets of eligibility criteria as follows: The non-opioid fibromyalgia group was required to not have taken any opioid medications within the last 90 days and not have taken opioid medications for >30 days in their lifetime. The opioid fibromyalgia group was required to have taken opioid medications for at least the past 90 days. Control participants were pain-free, free of any depression or anxiety disorder, and not taking pain or mood-altering medications. Data from the non-opioid and healthy control groups were analyzed and published previously (16).

**Table 1:** Demographic and clinical symptom measures are shown for each group with three group *t*-test comparisons between groups.

	**Healthy controls**			**FMN patients**			**FMO patients**			**HC vs. FMN**	**HC vs. FMO**	**FMN vs. FMO**
										***t*** **-test**	***t*** **-test**	***t*** **-test**
	***N***	**Mean**	**Std Dev**	***N***	**Mean**	**Std Dev**	***N***	**Mean**	**Std Dev**	***P*** **-value**	***P*** **-value**	***P*** **-value**
Age	14	48.71	11.10	15	47.13	9.82	15	53.27	6.73	0.687	0.200	0.056
WPI score	14	0.00	0.00	15	13.80	3.53	14	13.36	3.52	<0.001^*^	<0.001^*^	0.738
SS score	14	0.00	0.00	15	8.27	1.94	11	8.27	1.85	<0.001^*^	<0.001^*^	0.994
SHS	14	72.50	13.50	15	103.13	13.27	15	100.27	14.35	<0.001^*^	<0.001^*^	0.574
Fatigue	14	48.19	5.36	15	65.10	6.73	14	69.34	5.28	<0.001^*^	<0.001^*^	0.072
BPI severity	14	0.23	0.49	15	5.14	1.82	15	5.82	1.61	<0.001^*^	<0.001^*^	0.290
BPI interference	10	0.61	1.07	15	5.05	2.52	15	6.19	1.91	<0.001^*^	<0.001^*^	0.184

*Portions reused and modified with permission from Martucci et al. (16). BPI, brief pain inventory; HC, healthy controls; FMN, fibromyalgia non-opioid; FMO, fibromyalgia opioid; N, number of subjects, SHS, sensory hypersensitivity scale; SS, symptom severity; Std Dev, standard deviation; WPI, widespread pain index; P-value, significance (two-tailed independent samples t-test);*

* *p < 0.05*.

### Study Procedures

All procedures were approved by the Stanford University Institutional Review Board, were carried out in accordance with the approved protocols, and were conducted at the Stanford University Richard M. Lucas Center for Imaging. All participants signed written and informed consent acknowledging their willingness to participate in the study, understanding of all study procedures, and understanding that they were free to withdraw their study participation at any time.

Participants completed questionnaires including the Fibromyalgia 2011 Diagnostic Criteria (19) for Widespread Pain Index (WPI) and Symptom Severity (SS) scores, Fibromyalgia Assessment Questionnaire, the Sensory Hypersensitivity Scale (SHS) (20), the Short Form Brief Pain Inventory (BPI) (21), and PROMIS Fatigue (bank v1.0, adaptive) (22). The PROMIS Fatigue bank have been developed, calibrated, and validated in the general and diverse patient populations (M = 50, SD = 10) and are available for public use (www.healthmeasures.net/). Additional questionnaires and brain scans collected were not included in the present analysis.

### MRI Scanning Procedures

A 3T General Electric Signa Discovery MR750 scanner with a 16-channel head and neck neurovascular coil (GE Systems, Chicago, Illinois) was used for MRI scanning. The scan session included initial preparatory localizer scans, ASSET calibration scan, high-order shimming using the whole body coil of the scanner, a resting state functional scan, and structural scan sequences. The entire imaging session took 30–45 min. Participants were asked for verbal pain ratings via the scanner's intercom and their comfort was ensured throughout the MRI scanning session.

Functional magnetic resonance imaging (fMRI) scans of the cervical spinal cord were acquired using a 2D gradient-echo (GE) echo-planar-imaging (EPI) sequence [14 oblique slices, 4 mm slice thickness, repetition time (TR) 2,500 ms, interleaved acquisition, echo time (TE) 30 ms, flip angle 75°, FOV 160 × 160 mm^2^, with matrix size 128 × 128, and in-plane resolution 1.25 × 1.25 mm^2^, total of 264 volumes]. The field-of-view was centered at the middle of the C6 vertebra and extended from the most superior part of the C5 vertebra to the most inferior part of the C7 vertebra. One control subject's fMRI scan parameters differed slightly (flip angle 70°, 17 oblique slices, FOV 220 × 220 mm^2^ in-plane resolution 1.7 × 1.7 mm^2^, 3 mm slice thickness, 1 mm gap); exclusion of this subject did not change the results, therefore the final results include this subject's data. Verbal pain ratings (0–10 scale, with verbal descriptive anchors of “no pain” and “worst pain imaginable”) were obtained before and after the fMRI scans.

A high-resolution structural MRI scan was acquired using a single slab 3D fast spin-echo (FSE) T2-weighted sequence (Cube) [TR 2,500 ms, TE 85 ms (maximum), echo train length 70, slice thickness = 1.4 mm, FOV 240 × 240 mm^2^, matrix size 256 × 256, effective resolution 1.4 × 0.94 × 0.94 mm^3^, interpolated resolution 0.7 × 0.47 × 0.47 mm^3^, number of averages 2]. This scan was used for registration of fMRI images to the PAM50 T2-weighted spinal cord template De Leener et al. (23).

An additional structural scan with optimized spinal cord gray matter–white matter contrast was acquired using a 2D axial multi-echo recombined GE (MERGE) sequence (32 oblique slices, 3 mm slice thickness, 0.5 mm spacing, TR 525 ms, TE 5.4 ms, number of echoes 3, flip angle 20°, FOV 180 × 144 mm^2^, FOV centered at C6 vertebra, matrix size 320 × 192, in-plane resolution 0.35 × 0.35 mm^2^, and number of averages 2). The MERGE sequence images were used to assist with registration of internal spinal cord structures (i.e., gray vs. white matter) to the PAM50 template.

### Image Processing

We preprocessed the functional images as performed previously (24, 25) using customized in-house scripts, Oxford Center for Functional MRI of the Brain's (FMRIB) Software Library (FSL), and the Spinal Cord Toolbox version 3.0 (26, 27).

#### Motion Correction

We applied motion correction to the resting state fMRI data using a two-phase design calling FSL's Linear Image Registration Tool (FLIRT) with normalized correlation cost function and spline interpolation (28). First, a binary mask was manually drawn for each data set, which included the vertebral column, to create the reference image and exclude any regions of non-rigid motion from respiration and swallowing. In the first phase of motion correction, we used two-step 3D rigid body realignment: (1) We realigned the fMRI time series volumes to the middle time point reference volume, (2) we calculated the mean time series image and repeated realignment using the mean image new reference volume. In the second phase of motion correction, we used 2D rigid realignment to correct slice-independent motion; we realigned each axial slice independently using the mean image reference volume.

#### Registration to Template Space

We performed spatial registration to the PAM50 T2-weighted spinal cord template (resolution 0.5 × 0.5 × 0.5 mm^3^) to bring the fMRI images into the same image space (27). First, we cropped the T2-weighted structural image to include only C2 to T1 vertebrae. To create a structural segmentation mask, we segmented the spinal cord from the T2-weighted structural image. To create a vertebral landmarks mask, we marked the C2 and T1 vertebrae using the drawing feature in FSLview. Then, we straightened the T2-weighted structural image along the spinal cord using the structural segmentation mask and registered it to the template using the landmarks mask to guide registration along the superior-inferior (z) axis. Next, to initialize registration, we manually segmented the spinal cord from the MERGE (structural image with increased gray matter and white matter contrast) image, and used the template to T2-weighted image transformation to co-register the template to the MERGE image and the structural segmentation mask. Then, we segmented the spinal cord gray matter from the MERGE image, and used this to more precisely register the template to the internal spinal cord structures (i.e., white matter and gray matter). We then segmented the spinal cord from the mean motion-corrected fMRI image to create a functional segmentation mask, and co-registered the template to the mean fMRI image using the template to MERGE image transformation and functional segmentation masks to initialize the registration. This step was followed by non-linear registration in the axial plane only. Lastly, we concatenated the transformations from each of the above steps to allow for forward transformation of fMRI images to template space, as well as reverse transformation of the template masks into fMRI space (29).

#### Image Denoising

Spinal cord fMRI data are susceptible to noise from subject movement, cardiac and respiratory cycles, and cerebrospinal fluid (CSF) pulsations (30). To reduce the impact of physiological noise, we used FSL's physiological noise modeling (PNM) tool (31, 32) to create 16 slice-specific cardiac and respiratory noise regressors using physiologic data collected by the MRI scanner (sine and cosine terms with principal frequency and next three harmonics). Our rationale for this approach was based on retrospective correction of physiologic noise and motion effects (RETROICOR) (33) used previously in resting state spinal cord fMRI analysis (25). We generated additional multiplicative terms to account for interactions between the cardiac and respiratory cycles (total of 32 regressors). For CSF signal regression, we manually created CSF masks on the mean motion-corrected fMRI images and we used these to generate a slice-specific CSF noise regressor based on each slice's mean CSF signal. For white matter signal regression, as advised for spinal cord resting state fMRI analyses (34), we used the PAM50 template white matter probability map by warping to functional space, thresholding at 0.5 (≥50% probability), and eroding (to ensure no overlap with gray matter) to generate a slice-specific white matter noise regressor based on the mean white matter signal for each slice. In summary, our generated regressors included motion correction parameters (i.e., x, y, z rotations and translations from the first phase of motion correction), physiologic noise regressors (cardiac and respiratory), and tissue-specific noise (white matter and CSF). We regressed all of these from the motion-corrected fMRI time series using FSL's Improved Linear Model (FILM) (35).

#### Normalization of Functional Images, Spatial Smoothing, Quality Control

The denoised functional images were subsequently warped to the PAM50 template space. Spatial smoothing of the normalized fMRI images was performed with a 2 × 2 × 4 mm^3^ full-width half maximum (FWHM) gaussian kernel prior to the ALFF analyses. Lastly, visual inspection of the fMRI preprocessed data was performed for quality control.

### Mean ALFF Analysis

Amplitude of low frequency fluctuations (ALFF) is a measurement of low frequency oscillatory power based on the blood oxygen level dependent (BOLD) fMRI signal. It can be used as a general measure of CNS activity for resting-state fMRI data analysis. ALFF is advantageous for studying CNS activity because it is not dependent on the correlation of activity across selected regions of interest, but rather can provide independent measures of activity on a per-voxel basis. ALFF measures are calculated per subject and allow for comparison between patients and controls. It has been reported that ALFF has high test-retest reliability and, as compared with fractional ALFF (i.e., fALFF), ALFF has been shown to be more sensitive to individual and group-level differences (36). Thus, Mean ALFF was our primary measure to compare spinal cord resting state fMRI activity in patients with fibromyalgia taking vs. not taking opioids.

To calculate Mean ALFF for each voxel in each subject's preprocessed fMRI data, the Data Processing Assistant for Resting-State fMRI Advance Edition (DPARSFA) Toolbox (37) was used, running in Matlab R2015b on Windows 10 Pro. Initially, Mean ALFF was calculated across all low frequencies of 0.01–0.198 Hz and then tested for between-group differences. The Mean ALFF data were normalized (z-transformed) prior to statistical analysis.

To determine group differences in Mean ALFF, we analyzed normalized ALFF images to identify between-group differences using FSL's randomize tool. First, the normalized ALFF images (i.e., one per subject) were concatenated into a single multi-volume image. Then, the multi-volume image (i.e., 1 volume per subject) was processed using randomize to conduct a two-sample unpaired *t*-test of the images in each volume (38). Lastly, the significance level of between-group differences was evaluated with threshold-free cluster enhancement (TFCE) at both an uncorrected and corrected *p* < 0.05 (using 5,000 permutations).

### fMRI and Symptom Measures Correlation Analysis

For the correlation analyses of Mean ALFF values with symptom measures, we first extracted Mean ALFF values for each individual patient using regions of greater Mean ALFF and lesser Mean ALFF for frequencies 0.01–0.198 Hz (uncorrected *p* < 0.05) from the comparison of opioid and non-opioid patient groups. We then used these extracted Mean ALFF values to evaluate relationships with symptom measures across the two patient groups, and each patient group vs. healthy controls (IBM, SPSS Statistics, version 26). We included the following symptom measures from questionnaires in our analyses: average scan pain (mean of pre and post scan ratings), ACR fibromyalgia criteria widespread pain index (WPI) score, ACR fibromyalgia criteria symptom severity (SS) score, sensory hypersensitivity (SHS), fatigue (PROMIS Fatigue T-score metric, calculated using REDCap item response theory (IRT) scoring), pain severity (BPI), and pain interference (BPI). These symptom measures broadly represent sensory aspects of chronic pain (e.g., distribution of pain across the body, severity of other bodily symptoms, hypersensitivity to multiple types of sensory stimuli, sensation and experience of fatigue, pain intensity experienced on average, and pain interference experienced on average, respectively). The correlational analyses between fMRI data and questionnaire data were exploratory, selected for aspects of symptoms typically important for characterization of the clinical presentation of fibromyalgia (e.g., fatigue), and not corrected for multiple comparisons.

### Functional Connectivity and Graph Metrics Analysis

Functional connectivity is a measure of temporal correlation of signals between CNS regions, and provides a tool to understand the functional organization of brain networks and, more recently, spinal cord networks. Bilateral motor (i.e., right and left ventral horn) and bilateral sensory (i.e., right and left dorsal horn) resting state fMRI spinal cord networks exist (39–41) and have been shown to be altered after spinal cord injury and during thermal stimulation when applied unilaterally (25, 42). Therefore, because altered spinal cord processing may partially contribute to fibromyalgia, in the present study we also investigated spinal cord networks using functional connectivity analysis. We used a seed-based region of interest (ROI) approach as recently reported by our group (16, 25).

Functional connectivity strength was measured between left-ventral, left-dorsal, right-dorsal, and right-ventral horns at five levels (4.0 mm thick ROIs) which were vertically distributed (4.0 mm gap between ROIs) within the cervical spinal cord FOV (20 ROIs total). We generated the ROIs using the corresponding PAM50 spinal cord template probabilistic gray matter mask (0.5 thresholded) (23). We then extracted the mean time series from the preprocessed bandpass filtered (0.01–0.198 Hz) functional images for each ROI and for each participant, and created correlation matrices by calculating Fisher-transformed Pearson correlation coefficients between each ROI pair. We then calculated the mean ventral-ventral (V-V), dorsal-dorsal (D-D), ventral-dorsal within hemi-cord (V-D within), and ventral-dorsal between hemi-cord (V-D between) functional connectivity across the five levels. We compared functional connectivity strength to no connectivity (*r* = 0) within each group (one-sample *t*-test) and between groups (two-sample *t*-test).

### Graph Metrics Analysis

Lastly, we calculated weighted, undirected global graph metrics of modularity, efficiency, and small worldness to estimate topological properties of functional networks across the 20 ROIs. We calculated the graph metrics with GraphVar software and the Brain Connectivity Toolbox, and we used the absolute value of weights and relative thresholds over varied link densities (10, 20, 30, 40, and 50%) (43, 44). We normalized our graph metrics to metrics from 100 participant-specific random generated (5,000 iterations). We identified between group differences in graph metrics using a repeated measures general linear model (i.e., repeated measures ANOVA with between-group effects) across link densities for each of three group comparisons (1) healthy controls vs. non-opioid patients, (2) healthy controls vs. opioid patients, and (3) non-opioid patients vs. opioid patients.

## Results

Patients with fibromyalgia not taking opioids (*N* = 17), patients with fibromyalgia taking opioids (*N* = 16) and pain-free healthy controls (*N* = 17) participated in the study. We excluded data from six participants due to issues of poor resting-state fMRI image quality (one non-opioid patient, one opioid patient, two controls), scan artifacts (one non-opioid patient), and incorrect resting-state fMRI scanner sequence prescription (one control). Thus, we included in the analysis a total data set from 15 non-opioid patients, 15 opioid patients, and 14 healthy controls. Patients continued their usual prescribed non-opioid and opioid medications during the study (see Supplementary Table 1 and Supplementary Figure 1). Each patient underwent an MRI session of the cervical spinal cord (spanning the C5, C6, and C7 vertebrae) which included a fMRI scan to measure spinal cord activity at rest, and both sagittal and axial structural MRI scans for registration of the fMRI images to a standard template.

### Participant Symptom Measures

**Figure 1:**
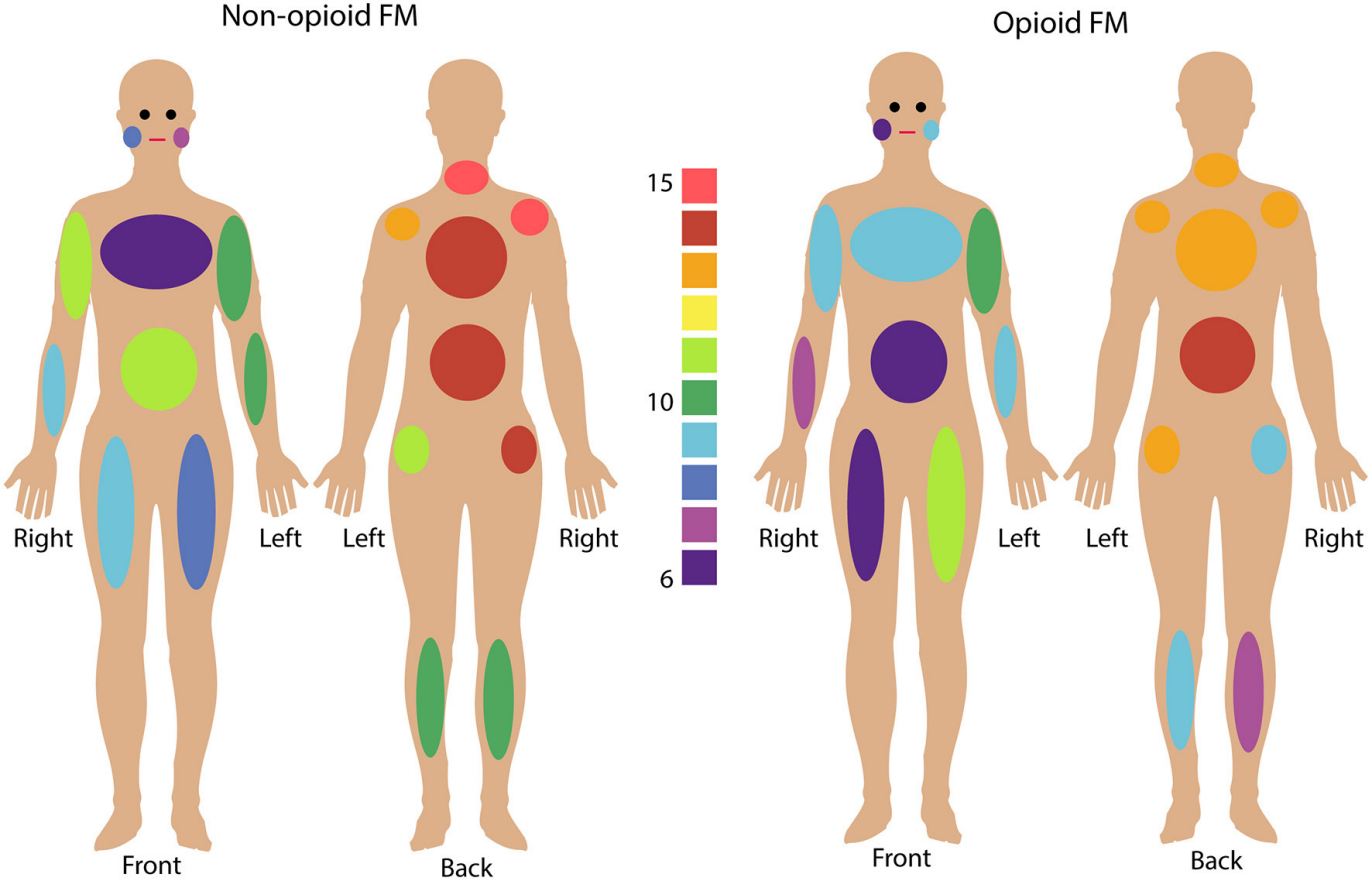
Fibromyalgia pain distribution across the body. Non-opioid patients (left) and opioid patients (right) are shown. Colors indicate the number of patients in each group who reported pain in any given body area using the Fibromyalgia Assessment Questionnaire. Bilateral body areas include right and left shoulder, upper arm, lower arm, hip, upper leg, lower leg, and jaw; and axial areas include chest, abdomen, upper back, lower back, and neck. (See Supplementary Table 5 for details on individual patient reported pain locations).

All participants reported their pain ratings before and after the fMRI scan and completed questionnaires measuring their pain distribution across the body, symptom severity, sensory hypersensitivity, and fatigue. Symptom measures of pain (WPI, SS, BPI), fatigue (PROMIS Fatigue, total converted T score), and sensory hypersensitivity (SHS) were greater in both opioid and non-opioid patient groups compared to the healthy control group, however, no symptom measures were significantly different between non-opioid and opioid groups (Table 1). Additionally, both opioid and non-opioid patients reported widespread pain distribution across the body, which is characteristic of fibromyalgia (Figure 1, individual patient details in Supplementary Table 5).

### Altered Regional Spinal Cord Mean ALFF in Opioid and Non-Opioid Patients

To characterize spinal cord activity in fibromyalgia patients who take opioids, resting-state fMRI images were analyzed and compared between groups of opioid patients and non-opioid patients, and healthy controls. The fMRI images were analyzed using standard preprocessing scripts and published methods to calculate the mean amplitude of low frequency fluctuations (ALFF) for each participant's data set. ALFF provides a measure of low frequency oscillatory activity (0.01–0.198 Hz) that occurs at rest in the CNS and is related to the BOLD fMRI signal.

**Figure 2:**
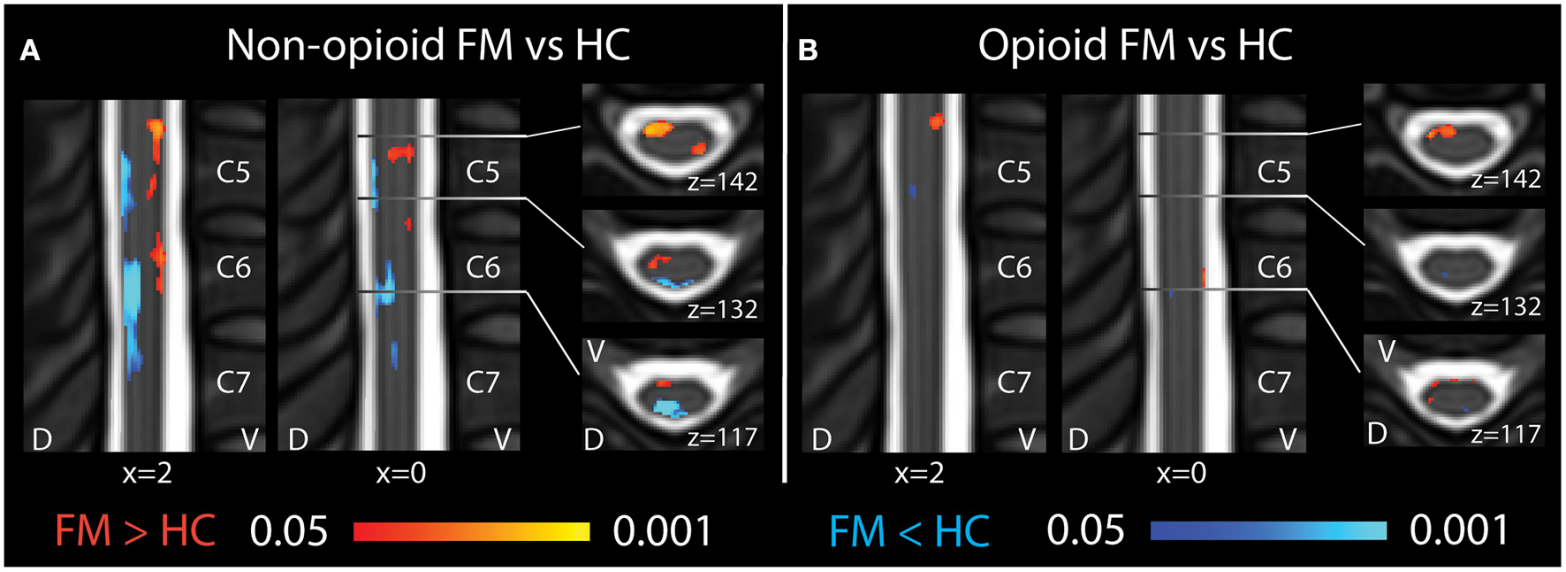
Spinal cord regional mean ALFF differences among patients taking and not taking opioids vs. healthy controls. **(A)** Compared to healthy controls, fibromyalgia patients who were not taking opioids (non-opioid FM) showed several spinal cord regions of ventral increases and dorsal decreases in Mean ALFF. Images reused and modified with permission from Martucci et al. (16). **(B)** Compared to healthy controls, opioid fibromyalgia patients (opioid FM) showed few regions of altered Mean ALFF. Red shading indicates regions of greater Mean ALFF and blue shading indicates regions of lesser Mean ALFF in patient groups vs. healthy controls. Sagittal images are indicated with “x” coordinate locations and axial images are indicated with “z” coordinate locations based on the PAM50 template (23). D, dorsal; FM, fibromyalgia; HC, healthy controls; L, left; R, right; V, ventral. *P*-values are uncorrected < 0.05.

**Figure 3:**
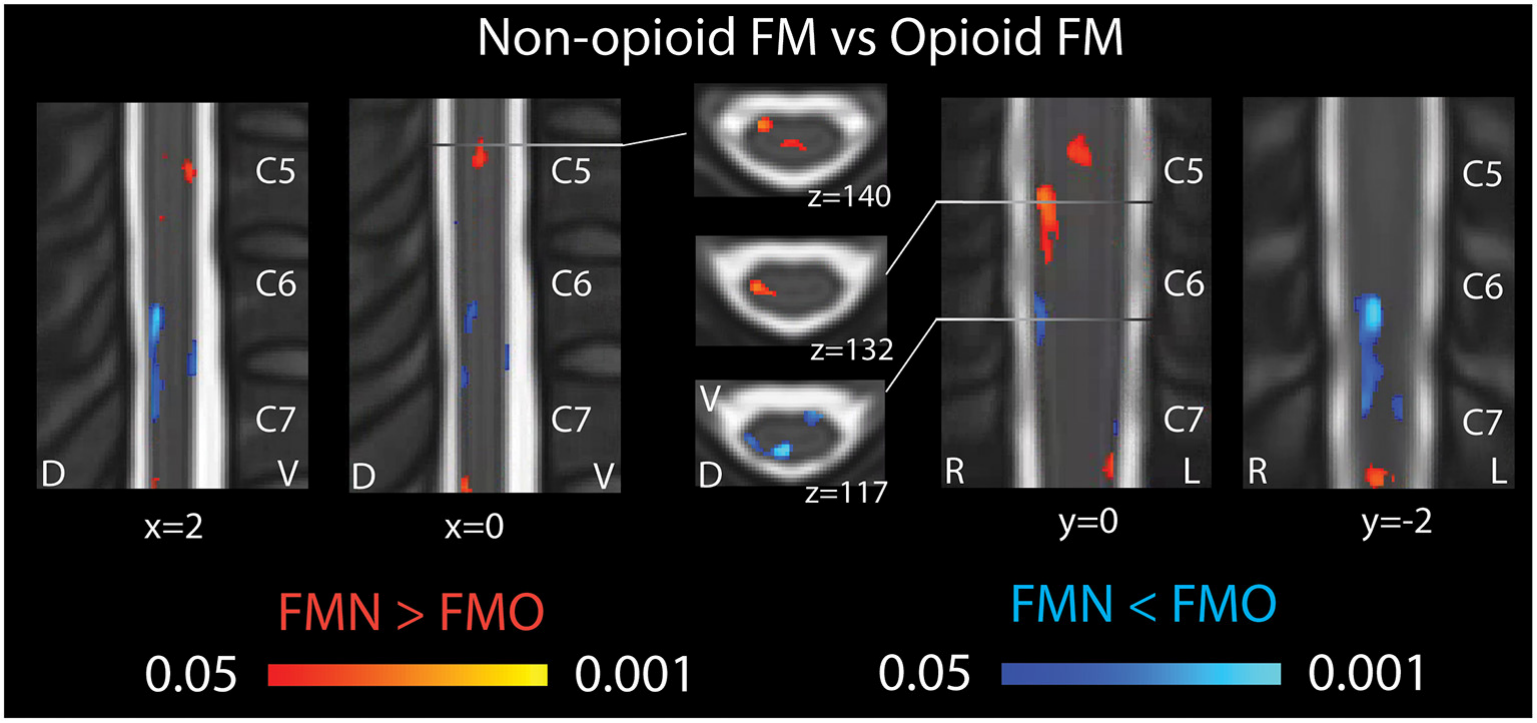
Spinal cord regional mean ALFF differences in patients not taking opioids vs. patients taking opioids. Fibromyalgia patients who were not taking opioids (non-opioid FM) showed several spinal cord regions of greater Mean ALFF vs. patients taking opioids (opioid FM) (regions of red shading, FMN > FMO). Non-opioid fibromyalgia patients showed several spinal cord regions of lesser Mean ALFF vs. patients taking opioids (regions of blue shading, FMN < FMO). Sagittal images are indicated with “x” coordinate locations, coronal images are indicated with y coordinate locations, and axial images are indicated with “z” coordinate locations based on the PAM50 template (23). D, dorsal; FM, fibromyalgia; FMN, non-opioid FM; FMO, opioid FM; L, left; R, right; V, ventral. *P*-values are uncorrected < 0.05.

In opioid patients as compared with healthy controls, we observed distributed regions of greater Mean ALFF and regions of lesser Mean ALFF, but only at the uncorrected threshold (uncorrected *p* < 0.05) (Figure 2). Comparing non-opioid patients to healthy controls, we observed more robust distributed regions of greater and lesser Mean ALFF (uncorrected *p* < 0.05), and a small region of greater Mean ALFF (corrected *p* < 0.05) [data published previously (16)] (Figure 2). Contrasting the two patient groups resulted in similar regional differences as observed between non-opioid patients and healthy controls (Figure 3). These regions overlapped with regional Mean ALFF group differences for non-opioid patients vs. healthy controls, and opioid patients vs. healthy controls. In summary, when comparing each patient group to healthy controls, opioid patients showed fewer regions of Mean ALFF differences than non-opioid patients (for cluster details see Supplementary Table 2). Additionally, individual patients' Mean ALFF values, extracted from non-opioid fibromyalgia greater than healthy controls (i.e., greater Mean ALFF, FMN > HC) vs. FMN < HC (i.e., lesser Mean ALFF) regions, were inversely correlated across patient groups (*N* = 30, Pearson correlation, *r* = −0.817, *p* < 0.001).

**Figure 4:**
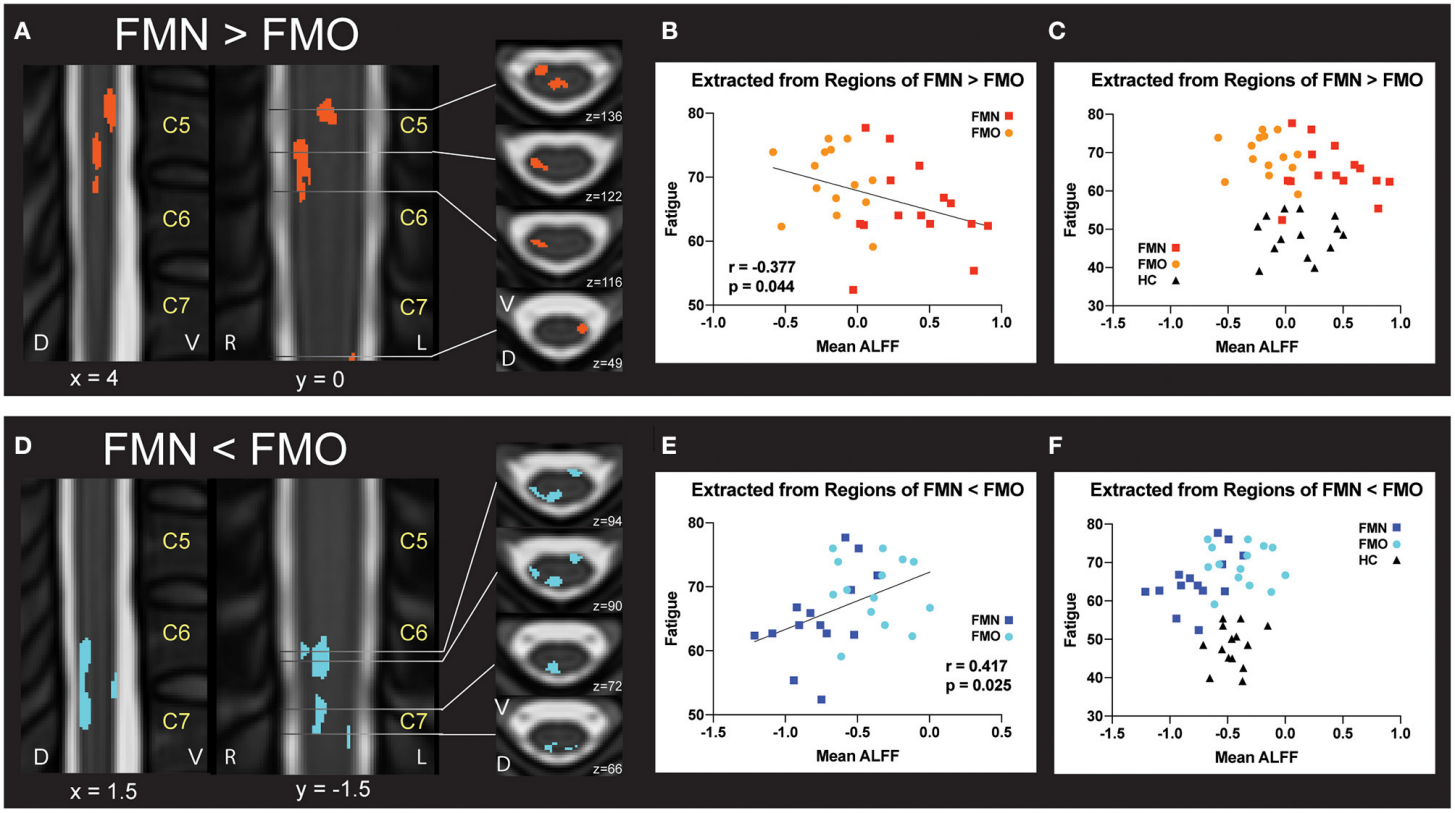
Correlations between regional mean ALFF and fatigue. **(A)** Regions of greater Mean ALFF were identified in fibromyalgia patients who were not taking opioids (non-opioid FM, FMN) vs. patients taking opioids (opioid FM, FMO). **(B)** Individually extracted regional Mean ALFF values were negatively correlated with fatigue across patient groups. **(C)** Healthy control values are plotted for visual comparison only. **(D)** Regions of lesser Mean ALFF were identified in fibromyalgia patients who were not taking opioids (non-opioid FM, FMN) vs. patients taking opioids (opioid FM, FMO). **(E)** Individually extracted regional Mean ALFF values from FMN < FMO regions were positively correlated with fatigue across patient groups. **(F)** Healthy control values are plotted for visual comparison only. Sagittal images are indicated with “x” coordinate locations, coronal images are indicated with “y” coordinate locations, and axial images are indicated with “z” coordinate locations based on the PAM50 template (23). ALFF, amplitude of low frequency fluctuations; D, dorsal; FM, fibromyalgia; FMN, non-opioid FM; FMO, opioid FM; HC, healthy controls, L, left; R, right; V, ventral. *P*-values are uncorrected < 0.05.

For the correlation analyses of Mean ALFF values with symptom measures, we first extracted Mean ALFF values for each individual patient using masks of the regions of greater Mean ALFF and lesser Mean ALFF for frequencies 0.01–0.198 Hz (uncorrected *p* < 0.05) from the comparison map of opioid and non-opioid patient groups (see Figure 4). We then used these extracted Mean ALFF values to evaluate relationships with symptom measures across the two patient groups, and all three groups. Across patient groups, fatigue (PROMIS Fatigue T score) positively correlated with Mean ALFF values extracted from regions of greater Mean ALFF in patients taking opioids; and fatigue negatively correlated with Mean ALFF values extracted from regions of lesser Mean ALFF in patients taking opioids (Figure 4). The relationships between Mean ALFF and fatigue were exploratory and not corrected for multiple comparisons. No other correlations of extracted Mean ALFF values were found with any other symptom measure (Table 2).

**Table 2:** Pearson correlations between region extracted Mean ALFF (0.01–0.198 Hz) and symptom measures across non-opioid FM patients (*N* = 15) and opioid FM patients (*N* = 15).

	**ALFF FMN** **>** **FMO**			**ALFF FMN** **<** **FMO**		
	***N***	***r***	***p***	***N***	***r***	***p***
Average scan pain	28	−0.074	0.708	28	0.155	0.431
WPI score	29	0.143	0.460	29	−0.185	0.337
SS score	26	−0.061	0.766	26	0.115	0.577
Sensory Hypersensitivity (SHS)	30	−0.112	0.556	30	−0.112	0.556
Fatigue (PROMIS)	29	0.417^*^	0.025	29	−0.377^*^	0.044
Pain Severity (BPI)	30	0.263	0.159	30	−0.189	0.318
Pain Interference (BPI)	29	0.196	0.308	29	−0.114	0.554

*ALFF, amplitude of low frequency fluctuations; BPI, brief pain inventory; PROMIS, patient-reported outcomes measurement information system; SS, symptom severity; SHS, sensory hypersensitivity scale; WPI, widespread pain index; r, Pearson correlation; p, significance (two-tailed).*

* *p < 0.05*.

We additionally conducted a *post-hoc* analysis, within the group of patients taking opioids, that identified no correlations between the Mean ALFF values and opioid dose (*r* = −0.015, *p* = 0.706; *r* = 0.023, *p* = 0.936; for values extracted from regions of greater and lesser Mean ALFF, respectively) (Supplementary Figure 2).

### Altered Functional Connectivity in Patients Taking Opioids

**Figure 5:**
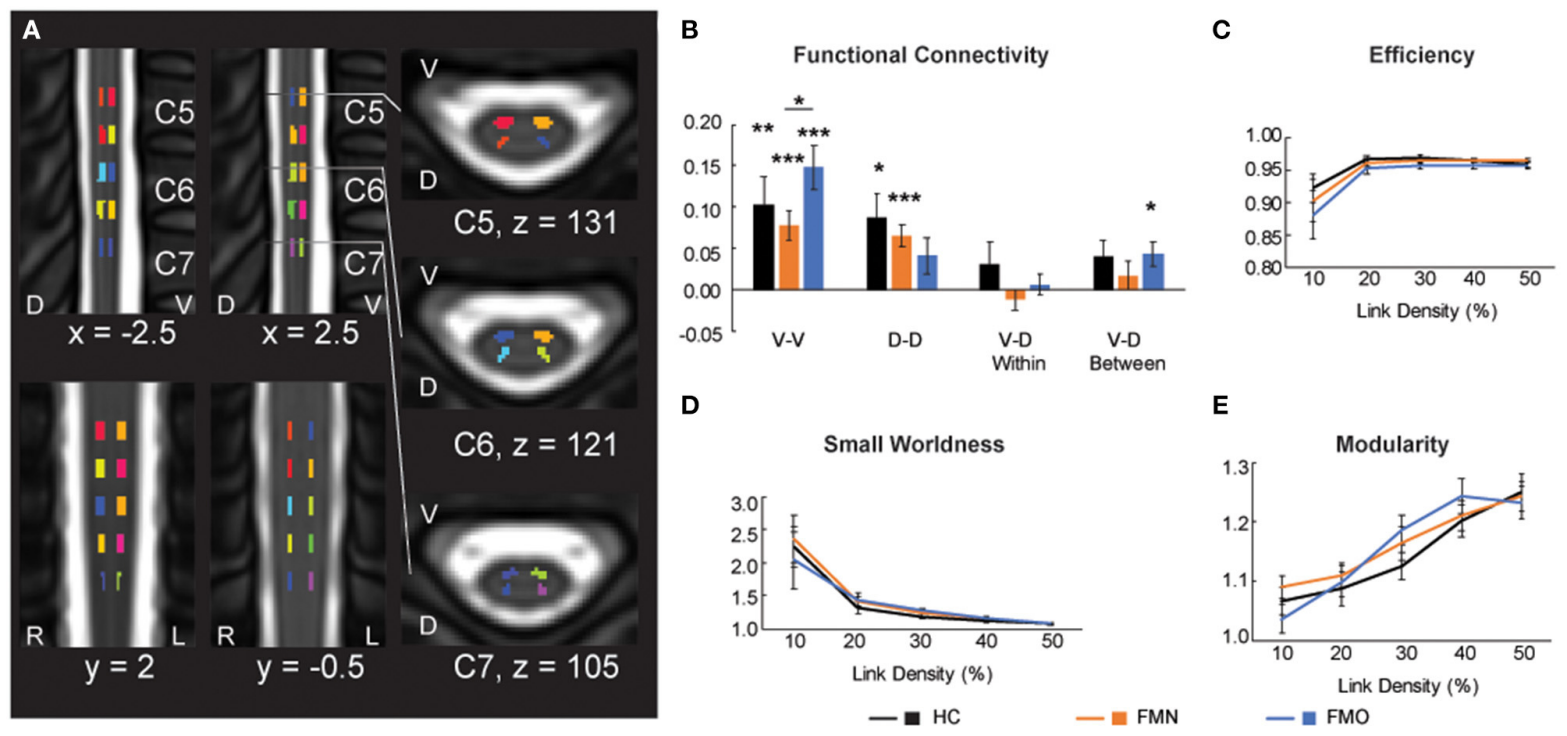
Functional connectivity and graph metrics. **(A)** Colored regions indicate spinal cord regions of interest that were used for the functional connectivity and graph metric analyses. Reused and modified with permission from Martucci et al. (16). **(B)** Functional connectivity for ventral-ventral (V-V), dorsal-dorsal (D-D), ventral-dorsal within (V-D Within), and ventral-dorsal between (V-D Between). **(C–E)** Graph metrics of efficiency, small worldness and modularity across the three groups: healthy controls (HC), fibromyalgia patients not taking opioids (FMN), and fibromyalgia patients taking opioids (FMO). D, dorsal; L, left; R, right; V, ventral. *p < 0.05; **p < 0.01; ***p < 0.001.

In addition to Mean ALFF values as a measure of regional activity within the spinal cord, we hypothesized that patients taking opioids would show altered functional connectivity between ventral and dorsal regions of the spinal cord. Bilateral motor (V-V) and sensory (D-D) functional spinal cord networks are found in healthy individuals (25, 39–41, 45). Thus, we analyzed mean ventral-ventral (V-V), dorsal-dorsal (D-D), ventral-dorsal within hemi-cord (V-D within), and ventral-dorsal between hemi-cord (V-D between) functional connectivity among the opioid patient group, non-opioid patient group, and healthy control group. We observed that V-V connectivity was significant (*r* > 0) for the opioid patient group (mean *r* ± 1 SE = 0.148 ± 0.026, *p* < 0.001), as well as for healthy controls (*r* = 0.104 ± 0.033, *p* = 0.008) and non-opioid patients (mean *r* ± 1 SE = 0.078 ± 0.018, *p* < 0.001). Additionally, we observed that D-D connectivity only trended toward significant for the opioid patient group (*r* = 0.041 ± 0.022, *p* = 0.075), and was significant for healthy controls (*r* = 0.087 ± 0.030, *p* = 0.013) and non-opioid patients (*r* = 0.065 ± 0.014, *p* < 0.001). We observed that V-D within connectivity was not significant for the opioid patient group (*r* = 0.006 ± 0.013, *p* = 0.629) nor other groups (healthy controls *r* = 0.031 ± 0.027, *p* = 0.266; non-opioid patients *r* = −0.012 ± 0.013, *p* = 0.360) (Figure 5). We observed that V-D between connectivity was significant for the opioid patient group (mean *r* ± 1 SE = 0.044 ± 0.015, *p* = 0.011), but was not significant for non-opioid patients (*r* = 0.017 ± 0.017, *p* = 0.325) nor healthy controls (*r* = 0.040 ± 0.021, *p* = 0.080). Please Note: Connectivity strength for non-opioid patients and healthy controls were reported previously (16) and are mentioned here for context in comparison to the opioid patient group.

We also tested for group differences in functional connectivity by comparing across each set of groups. Group differences between healthy controls vs. opioid patients were not significant for V-V (*p* = 0.302), D-D (*p* = 0.228), V-D within (*p* = 0.397), or V-D between (*p* = 0.873) functional connectivity. These results were similar to previously reported results of group differences between healthy controls vs. non-opioid patients which were not significant for V-V (*p* = 0.503), D-D (*p* = 0.514), V-D within (*p* = 0.149), or V-D between (*p* = 0.409) functional connectivity (16). The group difference between non-opioid patients vs. opioid patients was significant for V-V functional connectivity, with greater V-V functional connectivity for the opioid patient group (*p* = 0.038), but group differences were not significant for D-D (*p* = 0.362), V-D within (*p* = 0.318), or V-D between (*p* = 0.253) functional connectivity. Across the patient groups, no relationships were significant between functional connectivity measures and spinal cord Mean ALFF values (Supplementary Table 3).

### Unaltered Graph Metrics Among Patient and Healthy Control Groups

Graph metrics describe the topological properties of the connectivity of resting state functional networks (43), therefore, to evaluate opioid effects on spinal cord properties of connectivity we analyzed graph metrics of small worldness, efficiency, and modularity. Consistent with previous reports of graph metrics analysis (25, 46), we did not observe differences in small world properties at the lower link densities (all *p* > 0.05). Mean small worldness at the 10% link density was 2.240 ± 0.302 for healthy controls, 2.354 ± 0.365 for non-opioid patients, 2.038 ± 0.433 for opioid patients. We did not find any group differences to be significant (healthy controls vs. opioid patients *p* = 0.708; healthy controls vs. non-opioid patients *p* = 0.814; opioid vs. non-opioid patients *p* = 0.582). Additionally, we did not find any group differences across the range of link densities for graph metrics of small worldness, efficiency, and modularity (all *p* > 0.05) (Figure 5; Supplementary Table 4).

## Discussion

In the present study, we measured spinal cord resting-state fMRI-associated low frequency power, network functional connectivity, and graph metrics to compare spinal cord activity and networks in patients with fibromyalgia taking opioids, patients with fibromyalgia not taking opioids, and healthy controls. Importantly, our groups of patients with fibromyalgia taking vs. not taking opioids reported similar levels of pain and clinical symptoms. Despite this, we found that, compared to non-opioid patients vs. healthy controls, our opioid patients demonstrated fewer regional differences in spinal cord low frequency power (ALFF) compared to healthy controls. In addition, individual differences in regional Mean ALFF, across opioid and non-opioid patients, were correlated with self-reported levels of fatigue. Lastly, compared to the other groups, the opioid patient group showed slight differences in functional connectivity. Overall, compared to previously reported results contrasting non-opioid patients with fibromyalgia vs. healthy controls, patients with fibromyalgia taking opioids showed less altered spinal cord low frequency power, unique differences in functional connectivity, and these changes appeared to be related to self-reported fatigue.

Differences in ALFF in the patient groups were observed primarily as more activity in ventral regions and less activity in dorsal regions of the spinal cord. The regions of altered activity were predominantly within white matter regions of the spinal cord. These observed signal differences, while apparent in the white matter, could in fact, have occurred within the spinal cord gray matter because the BOLD fMRI signal may parallel spinal cord blood flow and diffuse outward from the center of the spinal cord. However, assuming that these activity differences occur predominantly in white matter, our results may relate to potential differences in transmission of sensory and pain-related information in patients not taking opioids, and to a lesser extent in patients taking opioids. The differences observed in ventral spinal cord were localized to regions of the right spinothalamic tract, which transmits thermal and pain-related information; thus, increased ventral activity in patients who were not taking opioids, and to some extent in patients who were taking opioids, indicates potential increased transmission of pain-related information in fibromyalgia. Conversely, the differences observed in the dorsal spinal cord were localized to regions of the dorsal columns / medial lemniscus, which transmit sensory, touch, and vibrotactile information; thus, decreased dorsal activity in patients not taking opioids, and to some extent in patients who were taking opioids, indicates potential decreased transmission of sensory information in fibromyalgia. Ultimately, these differences in patient groups suggest a potential imbalance in ventral-dorsal transmission of noxious and innocuous information, respectively. When simultaneous noxious and innocuous stimuli are administered to the skin, they inhibit the transmission of each other in the central nervous system (47, 48). Future studies including sensory testing experiments, such as thermal and vibrotactile stimuli, could help identify potential correlations with spinal cord activity, and support the hypothesis of imbalanced transmission of pain vs. sensory information in fibromyalgia, and how these changes may be influenced by opioid use.

Opioids exert their analgesic effects primarily via inhibitory, e.g., GABAergic, mechanisms, and this may explain the reduced observed differences in spinal cord activity in our patients taking opioids. Both patient groups (opioid and non-opioid) showed increased ventral spinal cord activity (i.e., Mean ALFF) vs. healthy controls, however, in the patients taking opioids the increased ventral spinal cord activity was much more limited. The limited increase in ventral spinal cord activity in patients taking opioids may represent opioid effects that would be expected to result in reduced transmission of pain-related information via the spinothalamic tract. The spinothalamic tract resides within the ventral spinal cord regions of observed activity differences in our results. Thus, our observation of less increased activity in the opioid patients is consistent with direct attenuation of responses of spinal nociceptive neurons (49), and indirect activation of descending supraspinal inhibition of noxious information by opioids (50). Further, opioids reduce brain response to noxious information, but not to vibrotactile information (51). Our findings similarly indicate that innocuous (e.g., vibrotactile) information transmission was not reduced by opioid use. Specifically, dorsal spinal cord activity (i.e., localized to dorsal column tracts that transmit innocuous/vibrotactile information) was minimally decreased in our opioid patients vs. healthy controls, but in contrast, dorsal spinal cord activity was markedly decreased in the non-opioid patients vs. healthy controls. Additionally, the minimally decreased dorsal spinal cord activity in the opioid group could be due to a secondary effect, whereby reduced pain transmission (as an effect of systemic opioid medication), in turn, enables increased transmission of sensory/vibrotactile information, via release of pain inhibition effects on sensory input (47, 52). More broadly, exogenous opioids inhibit primary nociceptive afferents, descending/ascending circuits, and downstream effects on supraspinal (brain and brainstem) targets (49). Indeed, differences in brain structure (7) and function (11) occur in chronic pain in the presence of opioids. Therefore, the group differences presently observed between opioid and non-opioid patients could be due to a wide-range of effects within the nervous system.

Across both patient groups, altered dorsal spinal cord ALFF positively correlated with self-reported fatigue, while ventral spinal cord ALFF negatively correlated with self-reported fatigue. These correlations between fatigue and spinal cord activity could relate to descending serotonergic drive or levels of metabolite concentrations in muscle tissue (e.g., protons, lactate, ATP), any of which could influence transmission of sensory and pain information within the spinal cord. For example, via descending spinal cord tracts, serotonin inhibits muscle afferents, which produces sensations of fatigue (53). Greater sensations of fatigue could also be produced by opioid-induced reductions in pain-related spinal cord activity, which thereby disinhibit transmission of sensory information, and increase serotonergic activity (54). Alternatively, increased metabolite concentrations in muscle tissue produce sensations of non-painful fatigue (55). Non-painful sensations of fatigue may be more prominent in the opioid patients due to reduced transmission of noxious information, allowing for disinhibited transmission of sensory information. Ultimately, the mechanisms underlying the relationships between fatigue and altered spinal cord activity in opioid and non-opioid fibromyalgia patients are complex and require further investigation.

### Limitations

There are several limitations to consider regarding our results. Overall, we found that patients taking opioids show less alterations in spinal cord activity vs. healthy controls (i.e., Mean ALFF) compared to patients not taking opioids (vs. the same healthy controls). Our findings are limited to fibromyalgia patients, within the observed ranges of pain severity, physical function, and psychological symptoms of the patients included in this study. Our patient groups reported similar levels of pain, depression, and anxiety, while a trend for greater fatigue was observed in the opioid patient group. Additionally, our groups sizes are modest in size and, due to the greater degree of noise inherent to spinal cord fMRI data, larger sample sizes (*N* = 20 or greater) should be used in future studies to replicate our present findings. Future studies with larger sample sizes would be expected to identify more robust group differences at corrected thresholds (i.e., our uncorrected threshold findings are purely voxel-wise and not corrected for multiple comparisons; they were calculated for each voxel using its individual distribution). Our findings are limited to the cervical spinal cord and future studies should determine if differences in spinal cord activity also exist in the thoracic and lumbar spinal cord of individuals with fibromyalgia.

In the cervical spinal cord, our findings suggest imbalanced ventral vs. dorsal activity observed primarily within the non-opioid patient group, and to a lesser degree in the opioid patient group. This ventral-dorsal activity imbalance may relate to greater transmission of noxious information and reduced transmission of innocuous information in fibromyalgia patients. The imbalanced ventral-dorsal activity was minimally apparent in the opioid patients, suggesting a partial normalization of this imbalance in patients taking opioids. This observed partial normalization in opioid-taking patients, is not consistent with our hypothesis that opioid patients would show greater altered activity due to mechanisms associated with opioid-induced hyperalgesia. However, the observed partial normalization could be due to opioidergic effects inhibiting transmission of noxious information, which in turn could also result in disinhibited transmission of sensory information. Similarly, while patients taking opioids had slightly higher levels of fatigue compared to patients not taking opioids, higher fatigue correlated with less altered spinal cord activity in the opioid group, which could be due to opioidergic inhibition of noxious information, thereby enabling more normal transmission of sensory, non-painful fatigue, sensations. However, the correlations identified in the present study need to be replicated and these posited underlying mechanisms should be tested empirically in future investigations with larger sample sizes. It is also possible that the apparent normalized activity may be due to compensatory mechanisms and may differ under conditions of sensory and/or painful stimulation; such hypotheses remain to be prospectively tested.

Ultimately, from the present investigation conducted during the resting state, we are not able to conclusively determine how analgesic (or other) effects of opioids relate to these pilot findings. Additionally in this study, both groups of patients were taking a variety of medications (see Supplementary Table 1) and some of the opioid patients were taking tramadol, which is a multimodal analgesic with opioidergic, serotonergic, and noradrenergic effects; these factors may have influenced our present results. It is also important to note that we did not control for timing of opioid dose and this may additionally contribute to variability in our findings (Supplementary Figure 1). In summary, due to the preliminary nature of this study, and the present lack of replication, our findings should not be used to draw clinical conclusions as to the appropriateness of using opioids in the treatment of fibromyalgia.

## Conclusion

In summary, our findings suggest that, compared to patients who do not take opioids, patients with fibromyalgia who take opioids show fewer alterations in spinal cord low frequency power and unique alterations in functional connectivity. These observed alterations in spinal cord activity may be related to opioid effects on spinal cord transmission of noxious vs. innocuous information and the experience of fatigue. It is hoped that future investigations building upon these preliminary and early findings may help us better understand the benefits vs. harms of long-term use of opioids in fibromyalgia, as well as help us understand the neurophysiologic effects of long-term opioid use for chronic pain.

## Data Availability Statement

The datasets generated for this study can be found in online repositories. The name of the repository and accession number can be found below: NeuroVault, https://identifiers.org/neurovault.collection:9624. The other raw data supporting the conclusions of this article will be made available by the authors, without undue reservation.

## Ethics Statement

The studies involving human participants were reviewed and approved by Stanford University Institutional Review Board. The patients/participants provided their written informed consent to participate in this study.

## Author Contributions

KM designed the study, collected the data, analyzed the data, wrote the manuscript, and edited and revised the manuscript. KW and SM provided assistance with study design, data analysis, and editing of the manuscript. All authors have read and approved the manuscript.

## Conflict of Interest

The authors declare that the research was conducted in the absence of any commercial or financial relationships that could be construed as a potential conflict of interest.

## Publisher's Note

All claims expressed in this article are solely those of the authors and do not necessarily represent those of their affiliated organizations, or those of the publisher, the editors and the reviewers. Any product that may be evaluated in this article, or claim that may be made by its manufacturer, is not guaranteed or endorsed by the publisher.
