# Supplementary material for: Spinal Cord Resting State Activity in Individuals With Fibromyalgia Who Take Opioids
Source: Front Neurol. 2021 Aug 4;12:694271. doi: 10.3389/fneur.2021.694271 (PMC8371264; doi:10.3389/fneur.2021.694271)
Supplement: Supplementary file 1 [file Data_Sheet_1.docx]

Supplementary Material

# Supplementary Tables


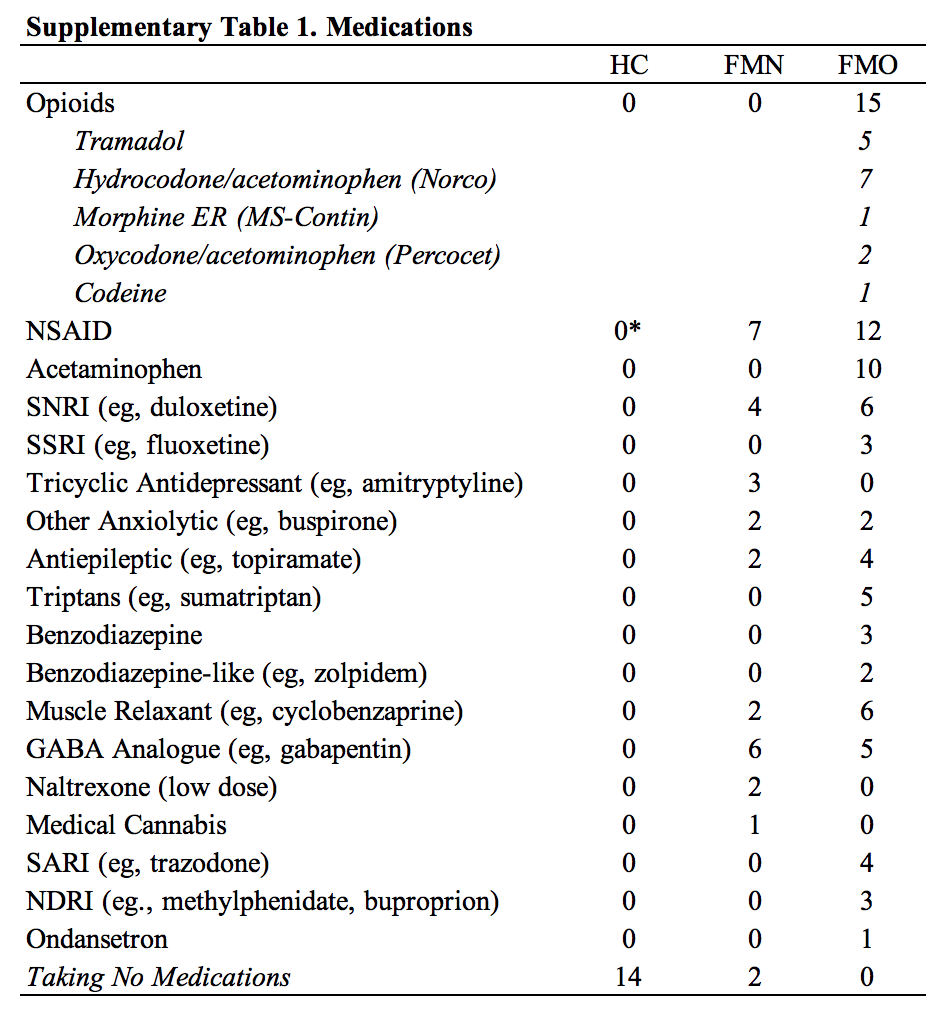


**Supplementary Table 1. Medications.** For each group of healthy controls (HC), fibromyalgia patients not taking opioids (FMN), and fibromyalgia patients taking opioids (FMO) the number of individuals in each group taking the class of medications is shown *Due to a sports-related ankle injury, one HC took celecoxib (200 mg) 3 weeks prior to the study visit. Gamma-aminobutyric acid, GABA, Nonsteriodal anti-inflammatory drug, NSAID; norephinephrine-dopamine reuptake inhibitor, NDRI; serotonin antagonist and reuptake inhibitor, SARI; serotonin and noradrenergic reuptake inhibitor, SNRI; selective serotonin reuptake inhibitor, SSRI.


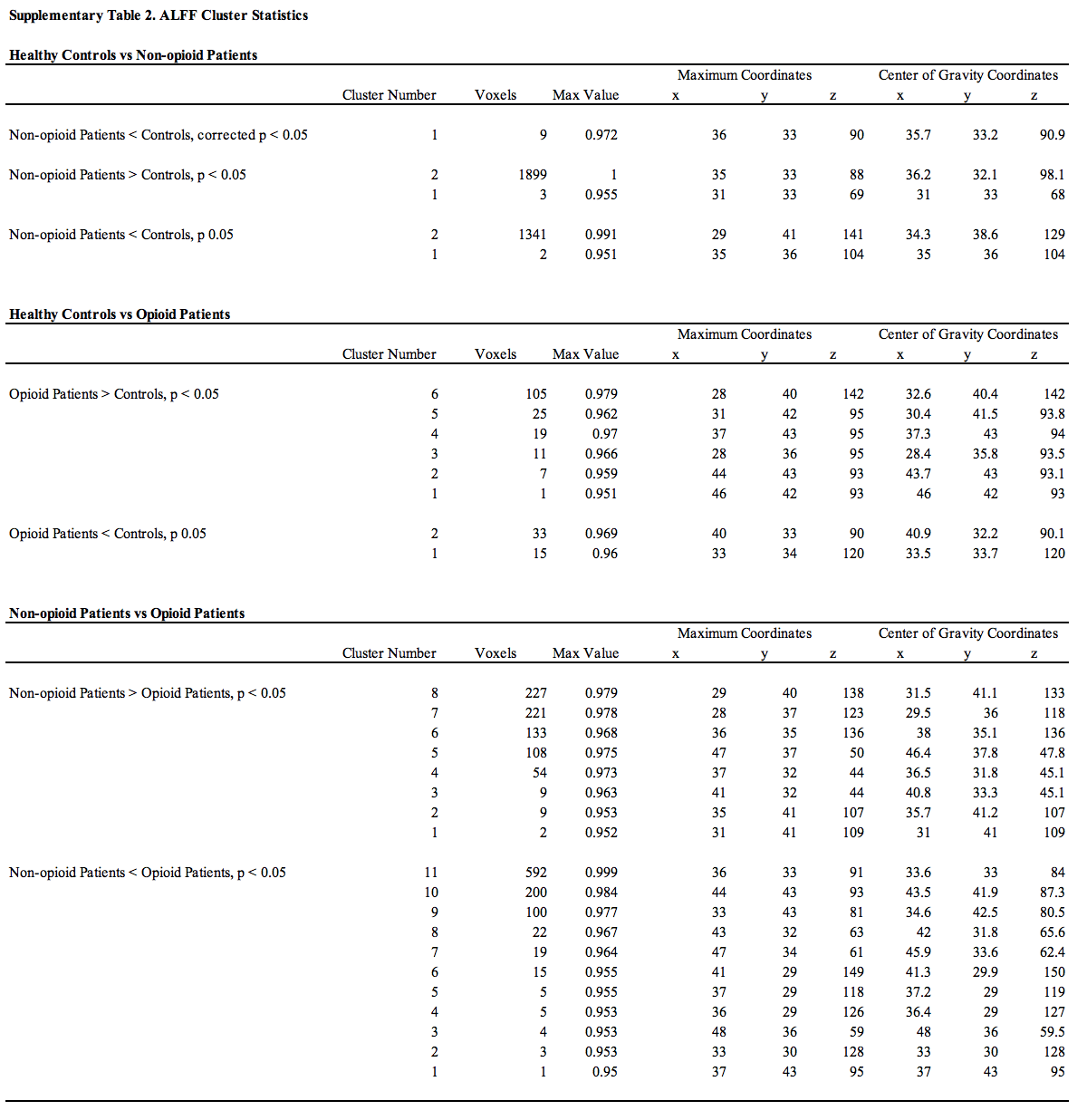


**Supplementary Table 2. ALFF Cluster Statistics.** Cluster location and statistics are shown for each comparison between groups of healthy controls, fibromyalgia patients not taking opioids (non-opioid patients), and fibromyalgia patients taking opioids (opioid patients).


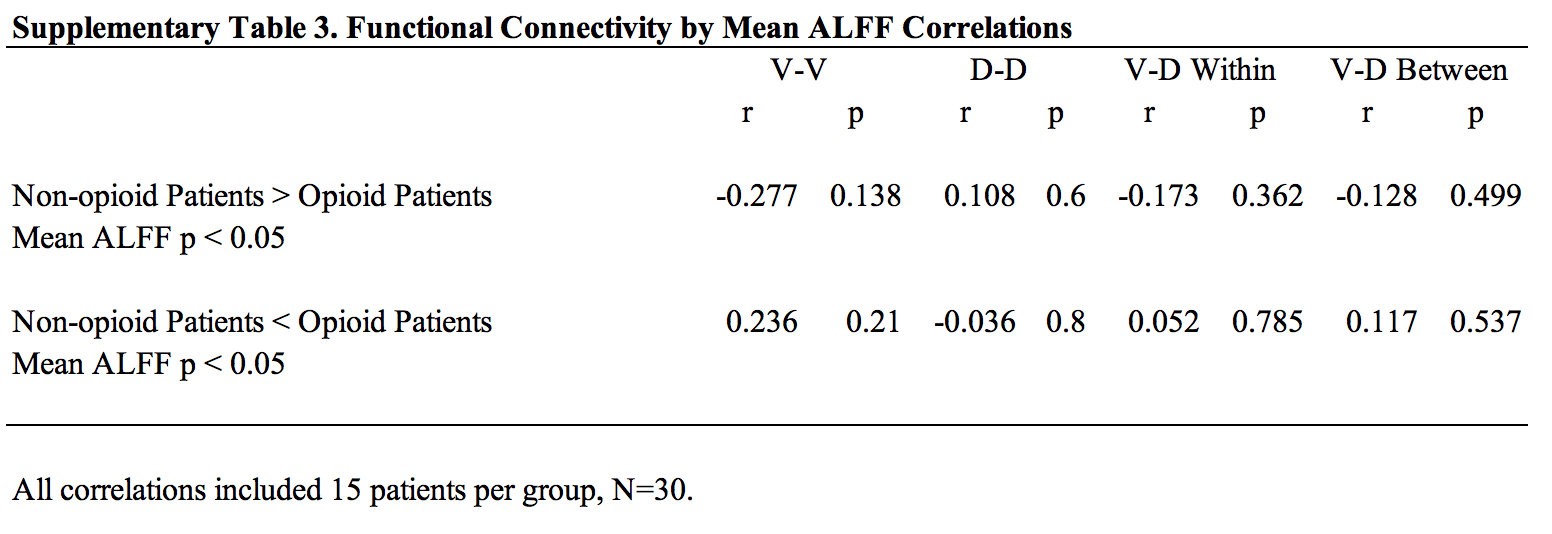


**Supplementary Table 3. Functional Connectivity by Mean ALFF Correlations.** Functional connectivity was tested for relationships with regional Mean ALFF differences between fibromyalgia patients not taking opioids (non-opioid patients) and fibromyalgia patients taking opioids (opioid patients). V-V, ventral-ventral; D-D, dorsal-dorsal; V-D Within, ventral-dorsal within hemicord regions; V-D Between, ventral-dorsal between hemicord regions.


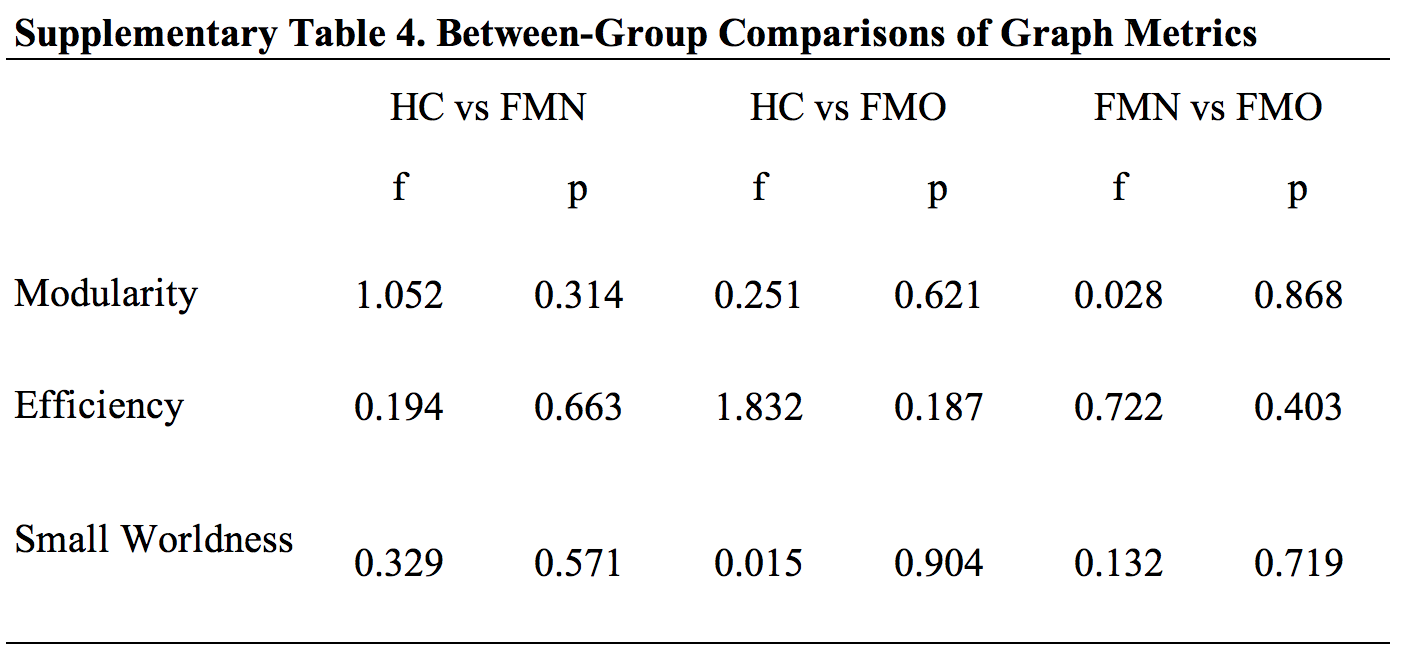


**Supplementary Table 4.** **Between-group Comparisons of Graph Metrics.** A repeated measures general linear model (i.e., rmANOVA for 5 factors due to 5 link densities per graph metric) was conducted for each group pairing (i.e., HC vs FMN, HC vs FMO, and FMN vs FMO) and each graph metric: modularity, efficiency, and small worldness. Between-group covariate effects are shown in the table for each group comparison. Abbreviations: HC, healthy controls (N=14); FMN, patients with fibromyalgia not taking opioids (N=15); FMO, patients with fibromyalgia taking opioids (N=15); p, significance (2-tailed).

**
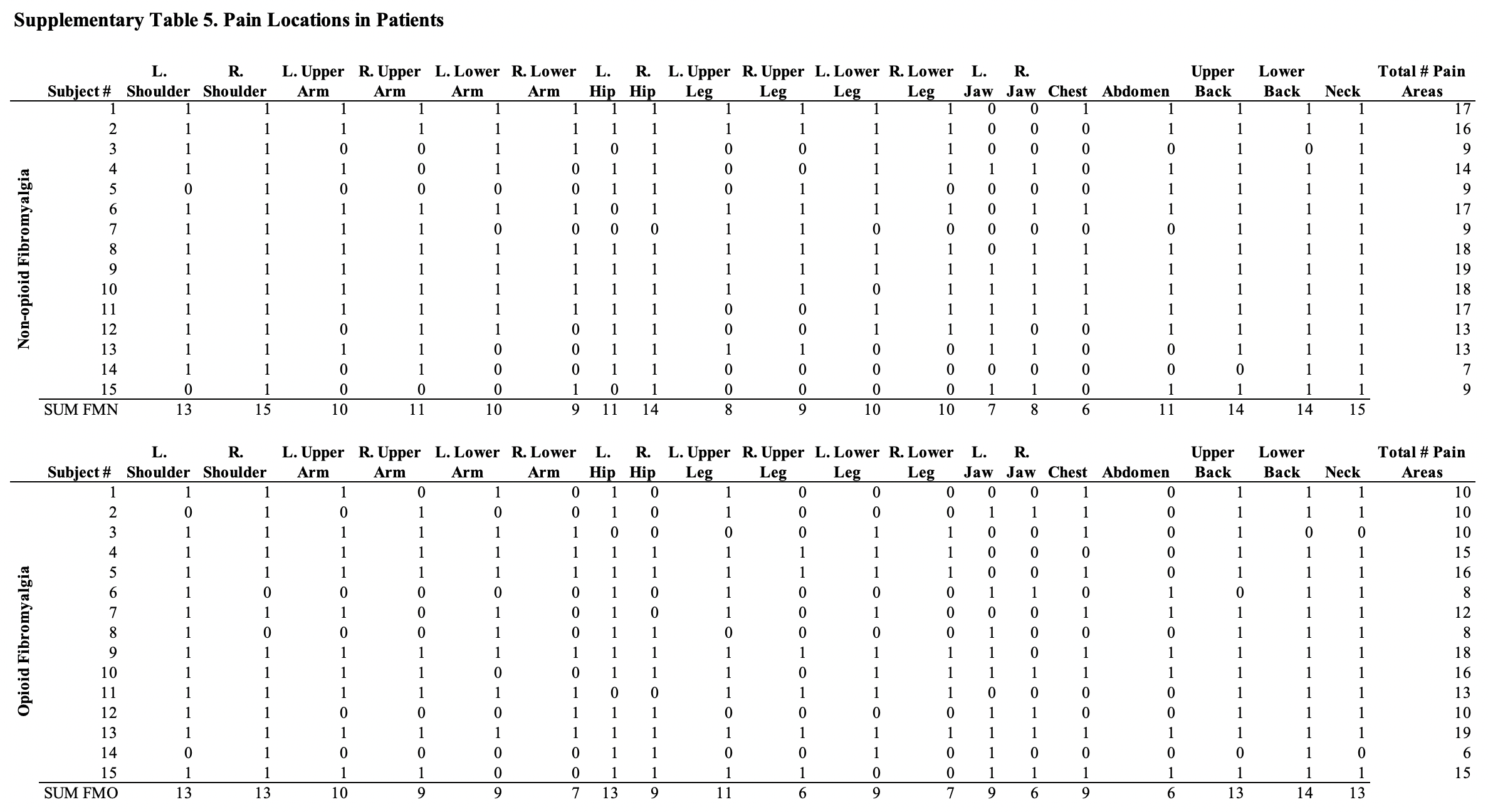
**

**Supplementary Table 5.** **Pain Locations in Patients.** Raw data collected to document pain locations across the body for individual patients in each group (see Fig. 1 for color map of group averaged pain locations). Abbreviations: FMN, patients with fibromyalgia not taking opioids “non-opioid fibromyalgia” (N=15); FMO, patients with fibromyalgia taking opioids “opioid fibromyalgia” (N=15); R., right; L., left; #, number.

# Supplementary Figures


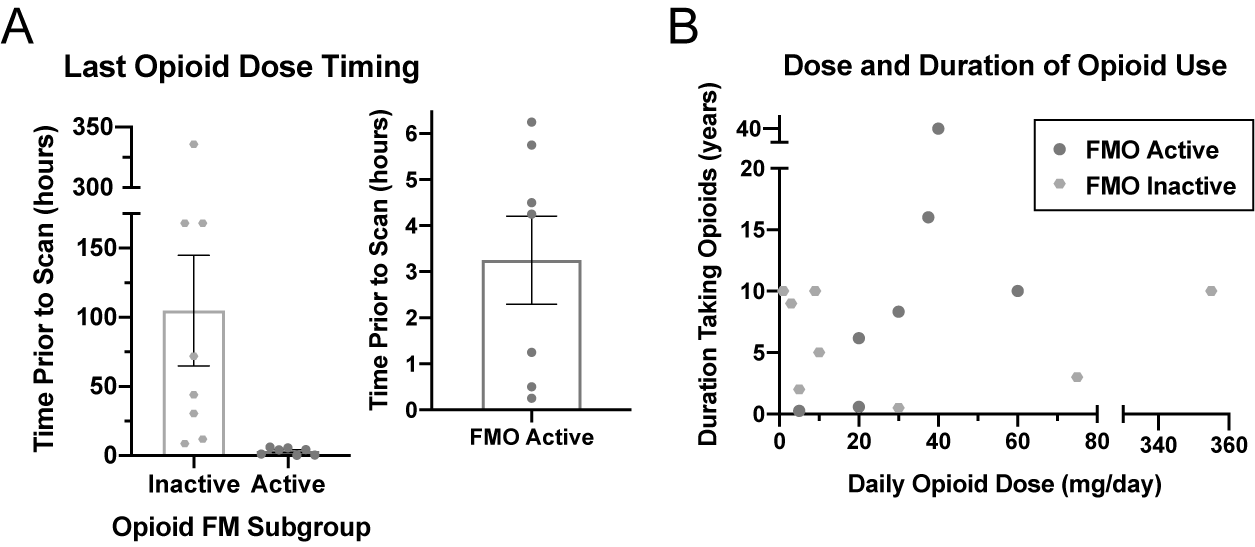


**Supplementary Figure 1.** **Opioid Dose Timing, Amount, and Duration of Opioid Use.**

**A)** Based on time of last opioid medication dose prior to the scan, patients are plotted as divided into subgroups of “active” opioid status for those whose last opioid dose was taken 6.5 hours or less prior to the scan (N=7), and of “inactive” opioid status for those whose last opioid dose was taken greater than 8 hours prior to the scan (N=8). (NB: Opioid status is not actual, but estimated based on standard medication metabolism.) Inset graph of “FMO Active” timing is provided to show details of variation in timing in the “active” opioid status group.

**B)** Variables of daily opioid dose (in morphine milliequivalents, MME was calculated using an online Opioid Dose Calculator (http://agencymeddirectors.wa.gov/calculator/dosecalculator.htm)”) are plotted against duration of opioid use for each patient (opioid-taking only). Shading indicates subgroup timing of last opioid dose prior to spinal cord fMRI scan data collection. Data are presented for descriptive purposes only, to characterize our opioid patient cohort. FM, fibromyalgia; FMO, patients with fibromyalgia taking opioids.


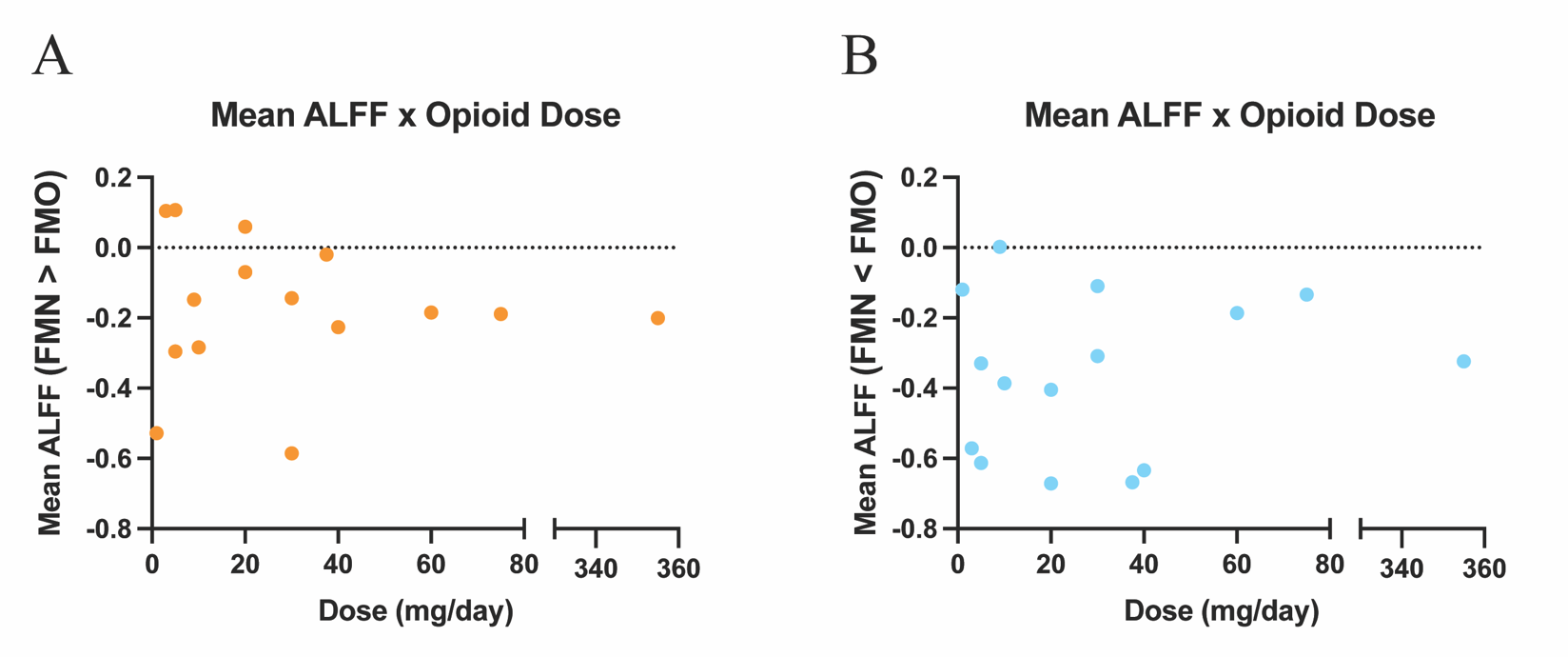


**Supplementary Figure 2. No Relationship Between Mean ALFF and** **Opioid Dose.**

In a post-hoc analysis, no correlations were observed between Mean ALFF values across individual patients taking opioids vs opioid dose. Mean ALFF values plotted were extracted from regions of Mean ALFF that were greater in FMN (**A,** r=-0.015, p=0.706**)** and lesser in FMN (**B,** r=0.023, p=0.936) as compared with FMO. Abbreviations: ALFF, amplitude of low frequency fluctuations; FMN, patients with fibromyalgia not taking opioids; FMO, patients with fibromyalgia taking opioids.
